# Quality in Higher Education and Satisfaction among Professors and Students

**DOI:** 10.3390/ejihpe11010017

**Published:** 2021-02-21

**Authors:** María del Carmen Olmos-Gómez, Mónica Luque-Suárez, Concetta Ferrara, Jesús Manuel Cuevas-Rincón

**Affiliations:** 1 Department of Research Methods and Diagnosis in Education, Faculty of Education and Sport Science, University of Granada, 52005 Melilla, Spain; mcolmos@ugr.es (M.d.C.O.-G.); jcuevas@ugr.es (J.M.C.-R.); 2 Department of Sociology, Faculty of Education and Sport Science, University of Granada, 52005 Melilla, Spain; 3 Associazione Culturale Italiana Per La Formazione, A.C.I.F. University of Naples, Via Salvatore Gambardella, 19, 80145 Napoli, Italy; concetta.ferrara@posta.istruzione.it

**Keywords:** educational quality, Student’s *t*-test analysis, satisfaction, higher education, innovation

## Abstract

The aim of this study was to analyze the significant differences in satisfaction with educational quality in higher education in Italy (Naples) among students and professors. The sample consisted of 501 higher education students and 121 professors, resulting in a total sample size of 622 subjects. Once the quality parameters of the instrument were determined, reliability was confirmed, and data collection was initiated. In order to analyze the results, a test of independent means (Student’s *t*-test) was performed, interrelating the variables of educational quality, concerning both management and satisfaction with higher education. Based on the results, we concluded that there are significant differences between the group of students and the group of professors, highlighting a higher level of satisfaction with quality reported by students regarding the coordination of teachers and staff in the educational process; therefore, the inclusion of students in the direct management of the center should be more active and an indicator to be taken into account in self-evaluation. Despite the limitations in the sample at the regional level, it offers many possibilities for future research.

## 1. Introduction

The world is changing, and these changes are determined not only by the society in which we live and grow up, but also by education, which therefore provides the means, together with other social aspects, to foster a global perspective in young people. These variations must be understood from a cooperative perspective, which makes it possible to transform the experiences and actions of students [1,2]. That is why it is necessary to guarantee quality in higher education institutions for social development [3].

López, Benedicto and León [4] consider that the educational system is important for the cohesion of society, as this cohesion requires quality, fair and equitable education for all. For education to exist, it is necessary that people actively participate in the educational system. To do this, it is necessary to define the next steps in research and in educational improvement, a task for which professors are responsible. This will allow the teaching/learning processes to address the challenges faced by 21st century colleges. However, these situations should be planned and verified considering the suitability of the proposed changes, so that students, professors and the educational institution itself are able to adapt to them [5].

When we deal with educational quality, this concept is regarded as a constant process of improvement to achieve general educational aims and goals [6], where the fundamental question is the degree of satisfaction with experiences within human development, knowledge, results, the resolution of conflict within higher education and the improvement of student well-being [7]. Authors such as Carvalho and de Oliveira Mota [8] indicate that institutions providing higher education should work to improve their efficiency and effectiveness, and thus achieve a general objective, which is the fulfilment of customers’ needs [9,10].

When evaluating teachers, authors such as Vincenzi [11] and Marciniak and Gairín [12] seek to analyze their perception of pedagogical training programs, study plans that can guide students, course design, didactic resources and classroom interaction. Authors such as Pimienta [13] consider that what is important in an evaluation that measures the educational quality of the teacher’s teaching activity is the opinion of those who directly receive the product of performance, i.e., the student body. According to Marsh [14], students are a source of evaluation of the effectiveness of the teacher’s performance and this can generate proposals for improvement at the level of educational quality control.

In the literature, there are studies that measure teacher effectiveness from the students’ perspective [13,15,16,17,18], although there are fewer studies that compare the perceptions of students and teachers together. Tobón [19] emphasizes that the emphasis is not on students, nor on teachers, but on the inter-systemic relationship of both.

Alvarado, Morales and Aguayo [20] have studied students’ perceptions of the quality of the services they receive from higher education centers based on factors such as physical infrastructure, the teaching process carried out by teachers and the ability to transmit knowledge, as well as the integral development fostered in students by academic programs, which is related to facilities, teaching materials, teacher qualifications, compliance with the course program and the integral development of the student, such as the willingness to help, extracurricular activities and orientation of the students’ professional future.

There are studies that address service quality in the education sector globally [21,22,23,24], of which the aim is continuous improvement, as a result of customers’ interaction with this sector. However, it is possible to find studies in which the concept of quality is presented in a confusing way and with different meanings [25].

Quality management refers to the implementation of a control system [26,27]. Some authors admit that such management covers many dimensions, and that is why it should be assessed at the same level as the established commitments [28,29,30].

Martinez, García and Quintanal [31] define the benefits of service-learning for the improvement of educational quality, and the achievement and maximization of the objectives as follows—professors will be able to innovate, within the context of education, which will allow students to build their own learning in an environment with real needs, the learning of skills will be facilitated and the learning and implementing of knowledge will foster the relationship and commitment with society. Authors such as Ledden et al. [32], Simpson [33] and Lago, López, Municio, Ospina and Vergara [34], describe students as consumers requiring a service from educational institutions, and the latter must meet its commitments, professors included. The higher the service quality, the higher the customer satisfaction [35,36].

In order to guarantee quality assurance, Bradbury [37] considers it important that the learning profile of each student is identified so that their individual needs are reflected and thus to work on one sole concept of quality in order to develop the formulation of proposals for improvements that will help maintain this cycle.

A new theory is being developed regarding quality management in education. The monitoring of the subjects is assessed, as well as the objectives to be met. External audits are performed to verify the degree of compliance with the set commitments, using management indicators, in order to be able to generate proposals for the improvement of future decisions that would help achieve the initially set objectives [38]. Students’ satisfaction with their education is very important in the assessment process, since it is becoming a key reference to distinguish quality from non-quality [39]. Likewise, the educational reforms take into account the level of satisfaction of the teaching staff and the management of the institution itself. Therefore, the most noteworthy commitment required to assess the level of quality of teaching has to do with the level of satisfaction of the people who are linked to the educational process.

In Italy, the country where our research was conducted, as in other countries, students are regarded as “consumers” and are evaluated on their performance. This generates a globalized competition which, in turn, emphasizes a move towards a more market-oriented approach in higher education institutions [40,41,42].

On the basis of the context described above, the main idea behind this study was to determine whether there are significant differences between the perceptions of professors and students regarding the quality and development of competence in institutions of higher education in Naples, as well as their satisfaction with the way they are managed.

## 2. Materials and Methods

### 2.1. Participants

For this research, a non-probabilistic sample was used, and the sampling used was incidental, casual, subjective or convenience sampling, since the subjects selected were those who were available, with prior bureaucratic permission, at the time the study was carried out. N = 622 total subjects, which consisted of 121 professors and 501 students, enrolled in different degrees and courses, from 16 out of 21 educational institutions in the eastern area of Naples, Italy. Regarding the age of respondents, the mean was 19.84 years (ages 17 to 25 years old) in the case of students. Concerning gender, 62% were females and 38% were males. The mean age of professors was 38.5 years, ranging from 35 to 45, with 51% being women and 49% being men. Relating socioeconomic status, 62.3% had medium socioeconomic status, 12.2% had low socioeconomic status and 25.5 % stated that to have a higher social status in the case of students. And in the case of professors 83.4% declared that to have a socioeconomic status medium, 6.2% had socioeconomic status low, and 10.4% stated that were on a high income.

### 2.2. Instrument

The participants took a Quality and Satisfaction Questionnaire [43,44,45,46], in paper format, which was previously validated and had proven reliability. The content validity of the instrument consisting of 45 items, was administered to a pilot sample (n = 439), with similar characteristics and were examined by seven experts in educational research using the Delphi method [47], through 3 rounds of analysis. The percentage of agreement in the final round was K = 91%. Construct validity was established with exploratory factor analysis. The Kaiser–Meyer–Olkin (KMO = 0.967) index was calculated. In addition, Bartlett’s test of sphericity was performed, showing a value that was significant at the 0.000 level. The result of this analysis explained by the Kaiser-Guttman criterion yielded 5 components to a total value of 70.75%. Lastly, criterion validity was established with model fit based on confirmatory factor analysis was satisfactory and yielded 4 factors; Parsimonious fit was (CMIN) = 832.6 (*p* < 0.001); Comparative Fit Index (CFI) = 0.87; Root Mean Square Error of Approximation (RMSEA) = 0.074 (90% CI; 0.053–0.080); Tucker-Lewis index (TLI) = 0.902. Regarding the QHES questionnaire, the reliability Cronbach’s alpha was good (α = 0.979), as well as the model fit [47,48,49]. Afterwards, it was coded and analyzed. The instrument was administered in public and private institutions, with 80% being public.

The questionnaire [43,44,45,46] consisted of 45 questions grouped into 4 sections, based on the dimensions identified by Olmos, Luque, Ferrara and Olmedo [45] in the Quality of Higher Education through the pursuit of Satisfaction, with a focus on sustainability (QHES). An initial section was added to these 4 sections (see Table A1 in Appendix A) which included identification and sociodemographic questions—group (student or professor), age, gender and socioeconomic status. For the answers to the 45 questions a coding system ranging from 1 to 5 (from “Strongly disagree” to “I always agree”) was used (see Table A2 in Appendix A).

### 2.3. Procedure

First of all, the heads of the educational institutions in Naples involved in the study were contacted, who granted permission to conduct the research. Professors and students were informed that participation in the study was voluntary and anonymous. Later, a paper-based questionnaire was administered to the students 25 minutes before the end of a class and the researchers were present throughout the whole process to clarify any doubts that arose. Data were collected during the first quarter of 2019. The study received the approval of the ethics committee in the Social Responsibility Committee at the University of Granada (code ML_19_3-19). Additionally, the study followed the ethical guidelines of the Helsinki Declaration.

### 2.4. Data Analysis

The psychometric properties of the instrument concerning validity and reliability were satisfactory [45,46,47,48,49]. Once the data were collected, the homogeneity of the sample was verified, reporting positive results for the parametric tests. Therefore, a *t*-test for two independent samples was performed as it was considered the most appropriate test for the comparison of the groups of professors and students. Likewise, and in accordance with the results obtained by Olmos, Luque, Ferrara and Cuevas [46], the 45 variables were divided into the 4 groups which demonstrated the highest validity. Data were analyzed using SPSS 24.0.

## 3. Results

Table 1 shows the results of the *t*-test, which was used as a data analysis technique to analyze the dependence and independence relations between the two variables. This test showed that there were significant differences with respect to the QHES questionnaire between the perception of professors and students in satisfaction among the five levels evaluated. This allowed for the observation of the effect of variance for independent means between the variables.

The results show (see Table 1) that the mean values differed between the dimensions of quality and satisfaction among professors and students. Significant differences were observed for leadership of academic resources in higher education, obtaining statistically significant differences for (F(degrees of freedom (df)) = 2.811, *p* < 0.05), and observing a greater average value in the group of professors (mean (M) = 4.32, standard deviation (SD) = 0.721) than in the group of students (M = 4.01, SD = 0.716). Significant differences were also observed for academic and administrative management of the planning of the teaching/learning curriculum (F(df) = 3.233, *p* < 0.05), with the group of students showing the lowest mean (M = 3.65, SD = 0.899), compared to that of professors (M = 3.82, SD = 1.131). This is one of the most noteworthy dimensions since it refers not only to the study program in which students indicated the need for improvement but also to knowledge and skill strategies. It is thus particularly important to take this aspect into account for improvement, development and innovation. Finally, significant differences have also been found in relation to the factor coordination of teachers and staff in the educational process (F(df) = 2.987, *p* < 0.05), with the highest level of satisfaction in this regard expressed by the students (M = 3.92, SD = 0.997), with a lower mean value in the professors’ group (M = 3.86, SD = 0.886).

## 4. Discussion

In this study, educational quality and the general level of satisfaction with this education have been analyzed through the opinions of higher education students and professors in a higher education community in Naples.

González [50] analyzed the dimensions of educational quality from the students’ perspective, through elements such as the enhancement of the skills needed to enter the labor force or students’ satisfaction with their access, on a constant basis, to up-to-date information relevant to the student population [51]. Proof of this are the results obtained after performing Student’s *t*-test, through which significant differences were found in three out of the four factors analyzed.

Regarding the first factor—leadership of academic resources in higher education, which integrates the items related to access to academic information, informative tutorials about the web pages of the institution, student counseling centers and the professional capacity of administrative staff—the group of professors expressed higher satisfaction with the access to academic information, with a significant difference of *p* < 0.005, whereas the group of students showed the lowest mean (4.01 < 4.32). Therefore, it is necessary to improve and increase accessibility to students through the platforms that they use in their daily practice [52], since the use of information technologies is essential in the daily life of students and the didactic resources and interaction in the classroom are essential for the correct perception of the students’ training [11,12].

The second dimension—planning of academic activities in university education—has to do with the organization of activities within the institution, complaint and suggestion forms at the educational level and the student environment in general within the facilities [53]. Concerning this factor, students were more satisfied with teaching activities and practices in general, whereas professors reported the lowest level of satisfaction. Although there were no significant differences and the percentages were similar, we can confirm that both groups were satisfied with the management of academic activities. Thus, the improvement of management in general, aiming to include the opinions of professors and their participation in the organization, could be a helpful measure for enhancing quality and their perception as professors, as a result of the inter-systemic relationship of teachers and students [19].

The third dimension—academic and administrative management of the planning of the teaching/learning curriculum—deals with all aspects related to the management of timetables, shifts and regulations [54,55,56]. Through greater involvement in the development and organization of higher education by students, who expressed significantly more dissatisfaction (3.65 < 3.82), management could help to improve this factor. Student participation in the center’s management, with the creation of seminars for the discussion and implementation of innovations from the students’ point of view, would improve the quality of the services that students receive in higher education centers [20].

Finally, the fourth dimension—coordination of teachers and staff in the educational process—covers the monitoring of subjects, the attainment of syllabus goals, tutorials and the creation of environmental expectations among students. The enhancement of this dimension, not only at the professor level but also at the student level, is essential for the improvement of teaching. Therefore, the promotion of technologies and innovation as teaching resources, as well as the development of continuous assessment and indicators of improvement of the curriculum and teaching development, are considered essential for posterity [57]. A significant difference of *p* < 0.005 was found in this area, with the teachers showing the highest mean (3.86 < 3.92), which reaffirms the first observation, namely, that the students make use of information technologies in their daily development. Therefore, the integration of information and communication technology (ICT) in the classroom, by counseling departments, academic institutions and the educational administration [58], such as the development of interactive programs, web page updates, online links, etc., are essential for the correct training of students [11,12].

It is worth highlighting students’ high level of satisfaction with the quality of facilities, as this implies, according to authors such as Vanacore and Pellegrino [59], who extended the work of Reference [60], a positive relationship between students and the institution where they are enrolled, generating a positive cognitive assessment and increasing their willingness to study.

## 5. Conclusions

In this study, we analyzed how the satisfaction of professors and students is an increasingly important factor in the teaching processes, skills and attitudes [61], as well as in education facilities [62]. Therefore, by researching educational quality, we are investing in the future.

Some authors [63] talk about the set quality standards that can be assessed by students, such as resources, academic and social aspects. Others have analyzed students’ perceptions of the quality of the physical infrastructures that guarantee the sustainability of better-quality education [64], and of the transmission of knowledge from professors and academic programs. Therefore, the study of the interaction between teachers and students must be carried out [19], and they should not be regarded as independent groups, since the improvement of quality depends not only on the management and infrastructure but also on the human factor and technological innovations.

Suggestions for improvements to this study include, first, increasing the sample size by including all the higher education institutions in southern Italy, with the aim of obtaining more significant results, and second, conducting new studies that provide evidence of the effects of satisfaction on the studied groups in relation to the variables of public or private education. Therefore, in conclusion, this research lays the groundwork for future multi-dimensional analyses.

## Figures and Tables

**Table 1:** Student’s *t*-test results sums of aggregated scales for the Quality of Higher Education through the pursuit of Satisfaction (QHES), comparing the groups of professors and students. M = mean, SD = standard deviation, CI = confidence interval, Sig. = significance.

						*t*-Test	
Factors		M	SD	CI (95%)		F	Sig.
				Lower Limit	Higher Limit		
Leadership of academic resources in higher education	Students	4.01	0.716	3.86	4.21	2.811	<0.005 *
	Professors	4.32	0.721	4.02	4.43		
Planning of academic activities in university education	Students	3.86	1.906	3.56	4.11	2.386	>0.005
	Professors	3.88	0.984	3.63	4.01		
Academic and administrative management of the planning of the teaching/learning curriculum	Students	3.65	0.899	3.23	4.03	3.233	<0.005 *
	Professors	3.82	1.131	3.62	4.09		
Coordination of teachers and staff in the educational process	Students	3.92	0.997	3.45	4.16	2.987	<0.005 *
	Professors	3.86	0.886	3.51	4.12		

Note: Adjustment was used for significance at 95% confidence level and below 0.005 *.

## Data Availability

The data presented in this study are available on request from the corresponding author.

## Appendix A

**Table A1:** Quality of Higher Education through the pursuit of Satisfaction with a focus on sustainability (QHES) questionnaire (identification and sociodemographic questions).

Section 1:	1. Sex: ___ Female/___ Male
Section 2:	2. How would you describe the socio-economic status of your family? ___ High ___ Medium ___ Low
Section 3:	3. Academic year: 1st ___ 2nd ___ 3rd ___ 4th ___ 5th ___ 6th ___
Section 4:	4. Groups: Students ___ Professor ___ Others ___

**Table A2:** Quality of Higher Education through the pursuit of Satisfaction with a focus on sustainability (QHES) questionnaire (45 questions).

	Strongly Disagree	Disagree	Unsure	Agree	I Always Agree
1. Management and teaching resources in higher education					
2. Coordination of educational activities in higher education					
3. Management of teaching content and staff in higher education					
4. Adequate timetables and shifts					
5. Rules in higher education					
6. Application of sanctions in higher education					
7. Communication between staff and parents					
8. Adequate timeline for achieving syllabus objectives (total duration)					
9. Existing procedures for filing complaints and / or offering suggestions with respect to teaching					
10. Overall satisfaction with higher education oversight					
11. Availability of syllabus information (web page or other sources)					
12. Accessibility of syllabus information (web page or other sources)					
13. Usefulness of existing syllabus information (web page or other sources)					
14. Orientation systems and welcome programs for new students.					
15. Objectives (skills) pursued by the syllabus					
16. Subjects reorient education to address sustainability—included on the syllabus					
17. Variety and adequacy of teaching methodology included in the syllabus					
18. Planned timeline to achieve syllabus objectives (duration of studies)					
19. Quantity of practical application included in the syllabus					
20. Sustainability activities as a complement to the overall formative development of the student					
21. Tutorials as a support system for better learning					
22. Support tutorials for students					
23. Collaboration between higher education and other sustainability civil society organizations					
24. Evaluation system used					
25. Expectations met by the syllabus					
26. Overall satisfaction with the syllabus					
27. Knowledge of subject matter of staff participating on syllabus courses					
28. Teaching skills and methodology of staff participating on syllabus courses					
29. Overall environment of cooperation and collaboration amongst students					
30. Public relations: degree of availability of staff to students					
31. Overall human environment: degree of availability of staff to parents					
32. Professional capacity of administrative staff					
33. Overall satisfaction with staff and respectful student environment within higher education					
34. Classrooms and equipment meet sustainability protocols					
35. Labs and workshops and their equipment meet sustainability protocols					
36. The library and its equipment					
37. Accessibility of the library					
38. Use of IT as a teaching resource					
39. IT lab use					
40. Sports facilities and equipment					
41. Existence of specific areas for protection of sustainability material and resources					
42. Catering services at the higher education institution					
43. Application of higher education sanctions					
44. Internet connection at the higher education institution					
45. Safety and hygiene when teaching					

Note: The variables worked with are extracted directly from the questionnaire QHES by Olmos, Luque, Ferrara and Olmedo [45].
